# Multiple infarcts and hemorrhages in the central nervous system of a dog with cerebral amyloid angiopathy: a case report

**DOI:** 10.1186/s12917-018-1700-0

**Published:** 2018-11-27

**Authors:** Laís Limeira Rodrigues, Leonardo Pereira Mesquita, Rafael Carneiro Costa, Raquel Gonçalves Gomes, Daniel Arrais Biihrer, Paulo César Maiorka

**Affiliations:** Departament of Pathology, Faculdade de Medicina Veterinária e Zootecnia, Universidade de São Paulo, Av. Prof Dr. Orlando Marques de Paiva, 87, São Paulo, 05508-270 Brazil

**Keywords:** Amyloid, CAA, Canine, Congophilic angiopathy, Ischemia, Stroke

## Abstract

**Background:**

β-amyloid (Aβ) can accumulate in the brain of aged dogs, and within vessels walls, the disease is called cerebral amyloid angiopathy (CAA). In humans, Alzheimer’s disease and CAA are strongly correlated with cerebrovascular disease. However, in dogs, this association has not been extensively studied yet. The present report highlights the pathological and clinical features of a concomitant cerebrovascular disease and amyloid precursor protein (APP) accumulation in the brain of a dog.

**Case presentation:**

A female, 16-year-old, Standard Poodle with a one-year history of cognitive deficits presented with an acute onset of right-sided postural reaction deficit and circling, left-sided head tilt, positional nystagmus, and ataxia. Due to poor prognosis the dog was euthanized, and pathological examination of the brain revealed an acute lacunar infarction within the thalamus extending to rostral colliculus. Additional findings included subacute and chronic areas of ischemia throughout the brain and areas of hemorrhage within the medulla. Immunolabeling revealed APP deposition within intraparenchymal vessels of frontal, temporal and occipital cortex, hippocampus, diencephalon, mesencephalon and myelencephalon, besides meningeal vessels walls. Glial fibrillary acidic protein (GFAP) immunolabeling showed marked astrocytosis around the acute area of infarction and within chronic areas of ischemia. Histological examination of the brain along with immunohistochemistry results showed a concomitant APP, which is an Aβ precursor, accumulation within the neuroparenchyma and vessels (CAA) with histological evidences of a cerebrovascular disease in an aged dog.

**Conclusions:**

This report shows that APP accumulation in the brain can occur concomitantly to a severe cerebrovascular disease in a dog. Further studies are necessary to elucidate if cerebrovascular disease is associated with Aβ accumulation in the brain of dogs.

## Background

Cerebrovascular disease (CVD), defined as any abnormality of the central nervous system (CNS) resulted from pathologic processes that compromises the blood supply [1], is a well-recognized cause of disability in humans. Despite its relative rarity, CVD is now recognized as a cause of neurological disfunction in dogs and cats and can be either classified as ischemic or hemorrhagic [2, 3]. Vessel occlusion by thromboembolism resulting in ischemic stroke and vessel rupture causing hemorrhage are the main direct consequences of CVD in dogs and cats [3]. Chronic hypertension, which results in atherosclerosis and lipohyalinosis in small penetrating arteries of the brain, is also suspected of being one of the main underlying conditions that may predispose dogs to CVD [4, 5]. Other predisposing conditions may include neoplasia, sepsis, hypothyroidism, parasites, vascular malformation, and coagulopathy [4].

Cerebral amyloid angiopathy (CAA), which is characterized by the deposition of β-amyloid peptide (Aβ) in parenchymal and leptomeningeal blood vessels, is a common disease in aged people, and associated with Alzheimer’s disease (AD) [6]. In dogs, a condition like AD, characterized by accumulation of senile plaques composed either by Aβ or Aβ precursor protein (APP), has been well described [7–12]. Importantly, the accumulation of Aβ within blood vessels (CAA), has also been reported in dogs [7, 10, 11]. In humans, CAA has been associated with vascular lesions, such as ischemic infarcts and intracerebral hemorrhages [6]. Intracerebral hemorrhages have also been reported in association with CAA in dogs [10].

Although infarcts, hemorrhages and white matter loss have been associated with CAA in humans [6], few studies have reported the occurrence of both brain infarcts and hemorrhages in dogs with CAA. The aim of the present study was to describe the clinical and pathological aspects of infarctions and hemorrhages within the CNS of a dog with APP deposition within vascular walls.

## Case presentation

A female, 16-year-old, Standard Poodle was presented at a private veterinary clinic. The owner reported several changes in the mental status 1 year prior to clinical examination. These changes were recognized as confusion and reduced awareness, impaired recognition of human family members and loss of previously learned abilities. Two days prior to clinical examination, the animal presented with a generalized tonic-clonic seizure. Subsequently, the animal presented with a poor appetite and lethargy, with circling and ataxia.

The neurologic exam revealed acute right-sided abnormalities characterized by postural reaction deficit, circling, and head and neck turn in addition to left-sided head tilt, positional nystagmus, and ataxia. The owner declined to perform a magnetic resonance imaging exam.

The clinical signs evolved to lateral recumbency and the dog was unable to feed itself, characterizing a poor prognosis. Therefore, the dog was euthanized and submitted for necropsy at the Department of Pathology of the School of Veterinary Medicine and Animal Science (FMVZ), University of São Paulo (USP). The brain was entirely fixed in 10% buffered formalin and routinely processed for histology. Sections of the brain were stained with hematoxylin and eosin, and were also submitted for immunohistochemistry for detection of APP and glial fibrillary acidic protein (GFAP). Briefly, several sections of CNS were submitted to antigen retrieval with citrate buffer pH 6.0. Then, the slides were incubated with primary antibodies anti-APP (Millipore, Darmstadt, Germany) and anti-GFAP (Dako, Agilent, Santa Clara, California, USA) diluted at 1:200 and 1:14000, respectively. The antigen-antibody binding was visualized using EnVision FLEX System kit (Dako, Agilent, Santa Clara, California, USA), according to manufacturer’s instructions. As a positive control for APP staining, a brain of dog with traumatic brain injury with axonal degeneration was used. A brain of a young dog without significant histological lesions were used as negative control for APP labeling.

Grossly, the lateral ventricles were mildly distended, with flattened gyri and mild widening of sulci.

**Fig. 1:**
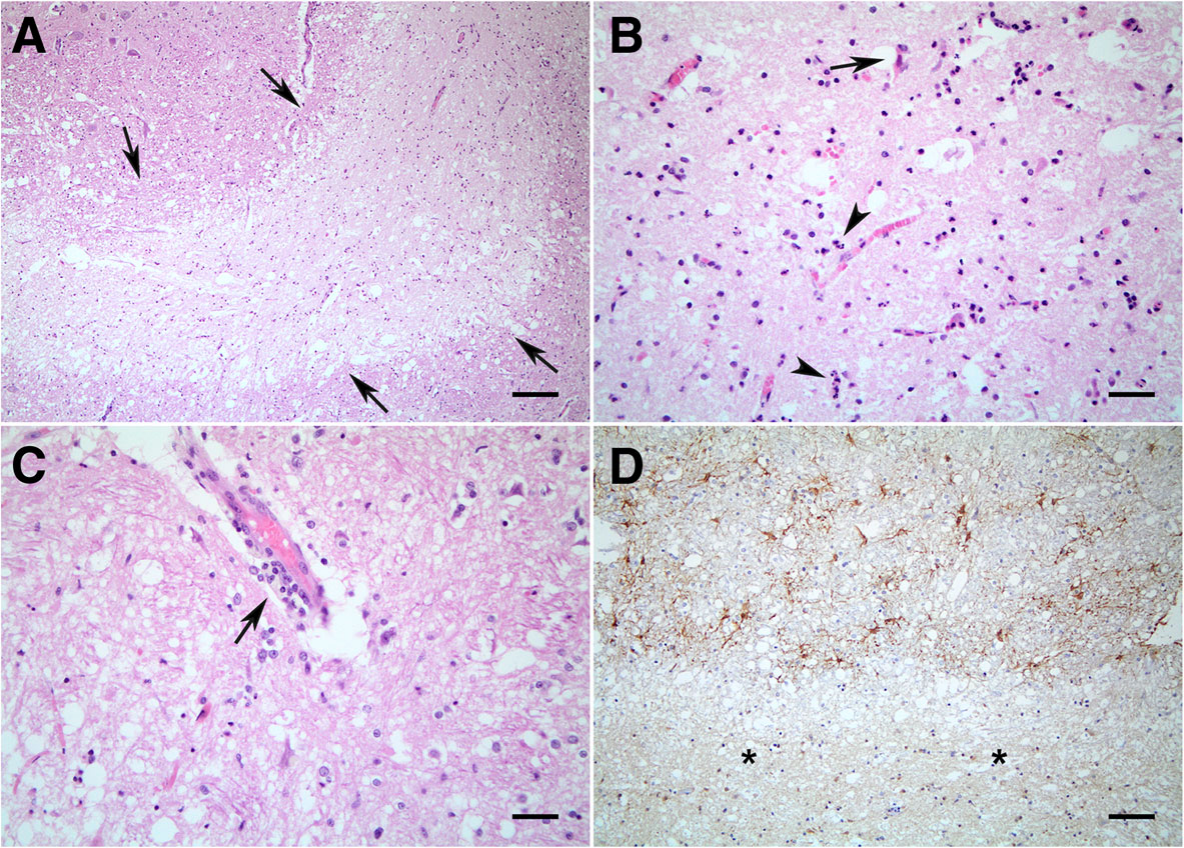
Cerebral amyloid angiopathy in a dog: central nervous system lesions. A-C, hematoxylin and eosin staining. **a**. At the level of oculomotor nucleus, a well demarcated, lacunar area of acute ischemia with coagulative necrosis surrounded by a rim of severe spongiosis (arrows) was visualized. **b**. Within the infarcted area there was neuronal necrosis (arrow) and degeneration and necrosis of glial cells (arrowheads). **c**. Perivascular cuffing of a low number of histiocytes was detected in vessels around the infarcted area. **d**. GFAP immunolabeling showed a severe astrogliosis (brown staining) surrounding the necrotic lesion (asterisks), in which astrocytic processes weren’t evident. Scale bars, **a** – 200 μm, **b** – 70 μm, **c** – 50 μm, **d** – 100 μm

Histologically, intraparenchymal and leptomeningeal blood vessels from many areas of CNS, including the myelencephalon up to cerebral cortex were mildly to severely thickened by an acellular and hyaline material. On the right paramedian region, extending from the thalamus, at the level of diencephalon and mesencephalon junction to the oculomotor nucleus, at the level of rostral colliculus, there was a locally extensive, well demarcated, lacunar area with marked loss of neuroparenchyma tinctorial properties with coagulative necrosis surrounded by a rim of severe spongiosis (Fig. 1a). Within the area of necrosis, multifocally, neurons had a pyknotic nuclei and hypereosinophilic cytoplasm (neuronal necrosis). There was also necrosis and degeneration of glial and endothelial cells, characterized by pyknotic and karyorrhectic nuclei (Fig. 1b). The perivascular compartments of surrounding vessels were infiltrated by a low number of histiocytes (Fig. 1c). Within the area of spongiosis there was a large number of swollen axons forming axonal spheroids (axonal degeneration). GFAP immunolabeling showed an increase in the number, complexity, and thickness of astrocytic process (astrogliosis) surrounding the necrotic lesion (Fig. 1d). Within the necrotic area, the astrocytic processes were not evident. GFAP was mildly detected within degenerated and necrotic astrocytes inside the necrotic area. These findings are compatible with an acute area of ischemia/infarction with initial reactive gliosis with minimal inflammation.

**Fig. 2:**
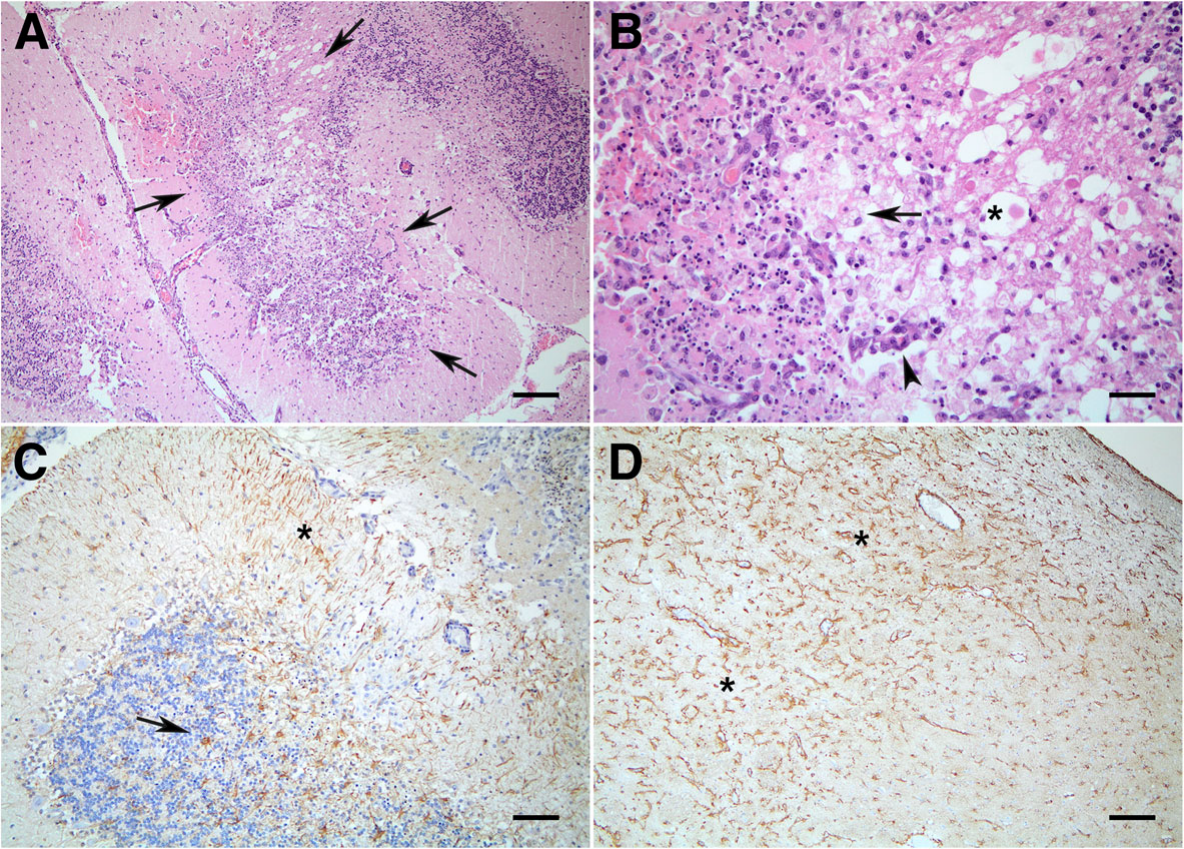
Cerebral amyloid angiopathy in a dog: central nervous system lesions. A-B, hematoxylin and eosin staining. **a**. In the cerebellum, a lacunar area of subacute infarction was visualized involving the granular layer and adjacent white matter (arrows). **b**. The subacute infarction was characterized by coagulative and liquefactive necrosis with Gitter cells (arrow), new-formed blood vessels (arrowhead) and axonal spheroids (asterisk). **c**. GFAP immunolabeling showed prominent astrogliosis (arrow), involving also the Bergman glia (asterisk). **d**. In chronic areas of ischemia there was a marked increase in the number of astrocytes (astrocytosis) and astrogliosis as demonstrated by GFAP immunolabeling (asterisks). Scale bars, **a**, **c**, **d** – 200 μm, **b** – 50 μm

Multifocally, lacunar areas of subacute infarction were visualized within the cerebellum, mainly involving the granular layer and adjacent white matter (Fig. 2a). The areas were characterized by a high number of granular cells with pyknotic and karyorrhectic nuclei, with liquefaction of neuropil, admixed with a moderate number of foam macrophages (Gitter cells) and axonal spheroids (Fig. 2b). These areas were surrounded by a moderate number of new-formed blood vessels with hypertrophied endothelial cells (neovascularization) with adjacent areas of hemorrhage and mild perivascular cuffing containing histiocytes. Prominent astrogliosis, demonstrated with GFAP immunolabeling, involving also the Bergman glia were visualized at the periphery of these necrotic areas (Fig. 2c).

Chronic lacunar areas of ischemia were also present within the telencephalon at the level of basal nuclei and hippocampus. These areas were pallor, well demarcated with neuron loss and with a marked proliferation of capillaries with hypertrophied endothelial cells. There was a mild to moderate spongiosis with an increase in the number of glial cells (gliosis), including a moderate number of cells with an abundant eosinophilic cytoplasm and an eccentric nuclei (gemistocytes). Rare perivascular cuffing of small number of histiocytes were visualized. Within these areas there was a marked increase in the number of astrocytes (astrocytosis) and astrogliosis as demonstrated by GFAP immunolabeling (Fig. 2d).

**Fig. 3:**
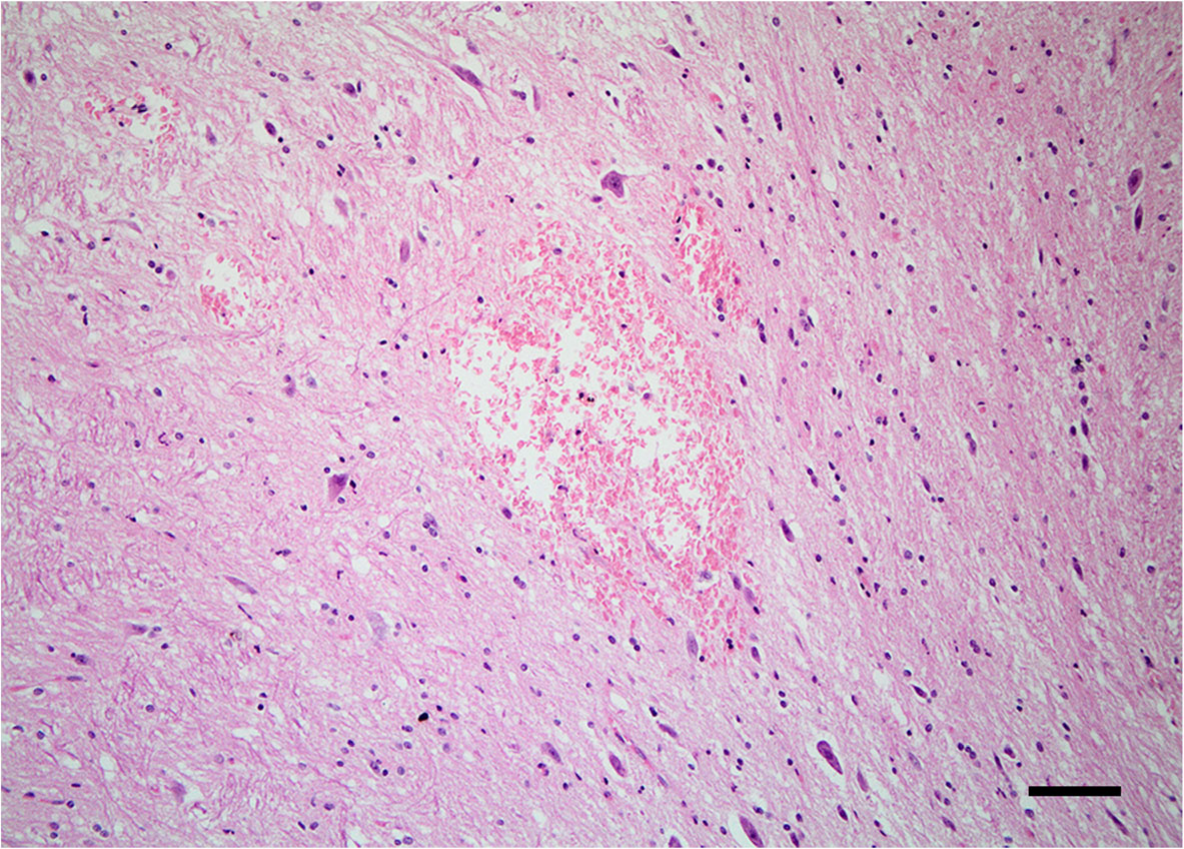
Cerebral amyloid angiopathy in a dog: central nervous system lesions. Hematoxylin and eosin staining. Multifocal hemorrhages within the medulla at the level of the obex. Scale bar, 100 μm

In additional to the areas of ischemia/infarcts, within the medulla at the level of obex, there were multifocal areas of hemorrhage within the reticular formation and several nuclei (Fig. 3). The white matter of many areas, including telencephalon to the myelencephalon, were variably degenerated.

**Fig. 4:**
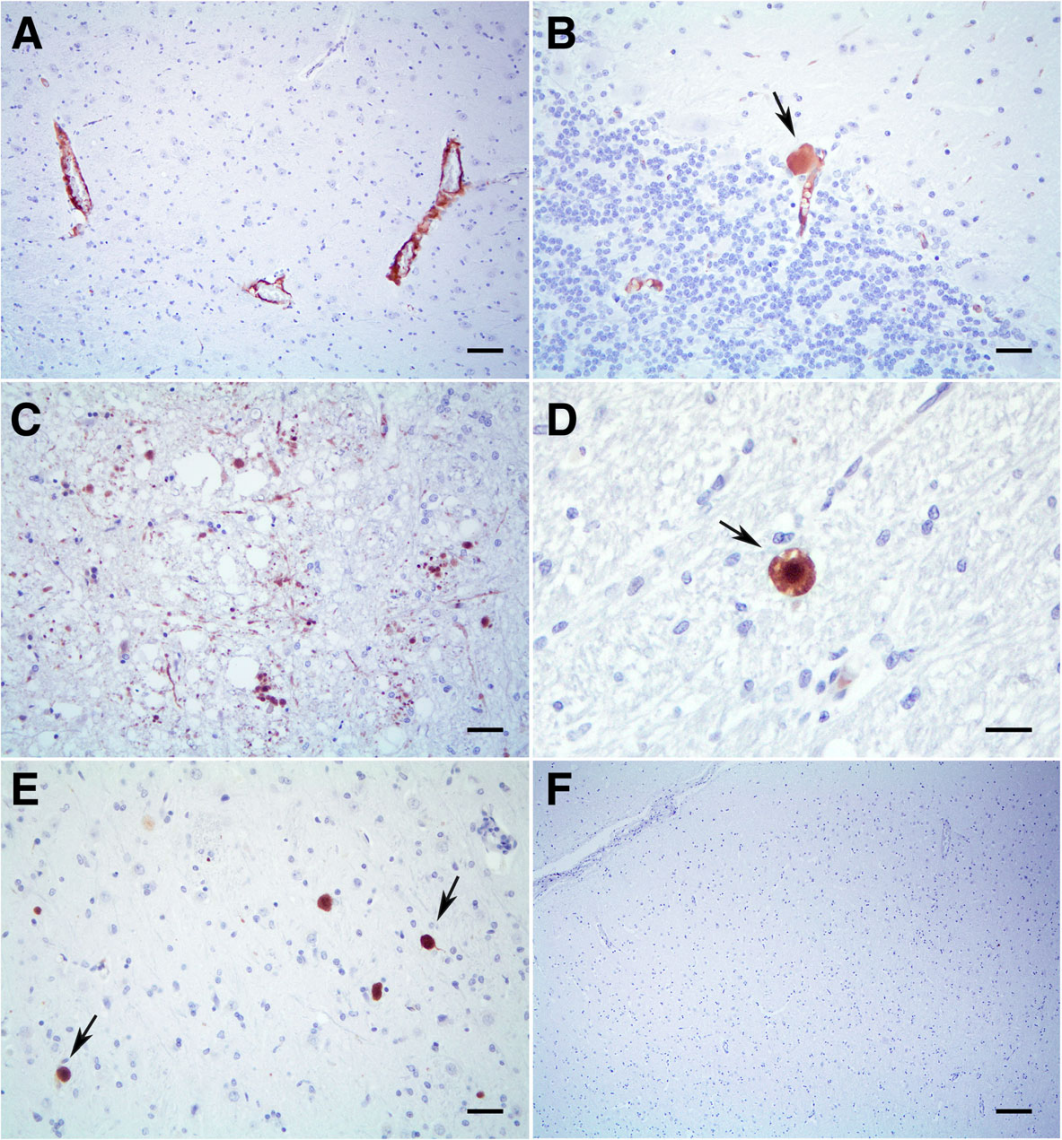
Cerebral amyloid angiopathy in a dog: detection of amyloid precursor protein (APP). **a**. The wall of intraparenchymal blood vessels was strongly immunolabeled for APP (brown staining). **b**. Additional spreading of APP from vessels to adjacent neuropil (arrow) of cerebellum was visualized. **c**. Swollen and degenerating axons were immunolabeled for APP within the periphery of the acute infarction. **d**. Small, up to 25 μm in diameter, areas of immunolabeling for APP containing a round, dense and darkly stained central region was detected. **e**. Other round areas of APP immunolabeling within the neuroparenchyma without a darkly stained central region was also detected. **f**. The brain of a control dog didn’t exhibit immunolabeling for APP. Scale bars, **a** – 70 μm, **b**, **c**, **e** – 50 μm, **d** – 50 μm, **f** – 200 μm

APP immunolabeling were visualized more frequently within the wall of intraparenchymal blood vessels (Fig. 4a), and occasionally affecting leptomeningeal vessels. Intensity of APP immunolabeling varied from mild to severe, according to Attems [6]. APP affected vessels from many areas of the CNS, but had a patchy distribution, characterized by APP-positive areas with adjacent APP-negative areas. Frontal lobes and temporal lobes were more affected when compared to occipital lobes. Other affected areas included the hippocampus, diencephalon, mesencephalon and myelencephalon. The cerebellum was less affected. Rarely, vessels with dyschoric change were observed, which consists in additional spreading of APP from vessels to adjacent neuropil (Fig. 4b). Importantly, a large number of hyalinized vessels weren’t immunolabeled for APP, especially those with a higher wall thickness. Within the periphery of the acute infarction, swollen and degenerating axons were immunolabeled for APP (Fig. 4c).

At the level of rostral commissure (basal nuclei) and thalamus there are locally extensive areas where a multifocal staining for APP was visualized within the neuroparenchyma. Within these areas, two types of APP staining were seen. Small, up to 25 μm in diameter, areas of staining containing a round, dense and darkly stained central region (Fig. 4d) were visualized in addition to other labeled areas, in which a central darkly stained core was not present (Fig. 4e) [13].

The brain of a young dog used as negative control didn’t exhibit APP immunostaining within CNS vessels or within the neuroparenchyma (Fig. 4f).

## Discussion and conclusions

The diagnosis of CAA was based on histological changes observed in blood vessels, and by demonstration of APP by immunohistochemistry within the wall of these vessels. In dogs, similarly as in humans, Aβ is derived from APP [14]. Therefore, in the present study, the detection of APP highly suggests an ongoing cerebrovascular disease associated with Aβ deposition. Besides vascular walls, APP immunolabeling was also visualized within the neuroparenchyma in some areas of the CNS in the dog from the present study. Importantly, this dog had histological lesions related to vascular injury, such as areas of ischemia/infarctions and hemorrhages.

Several neurological changes, like those observed in humans have been described in the CNS of old dogs (reviewed by Vite and Head [15]). These pathological changes, among others, includes Aβ deposits, CAA, vascular disease, and infarcts [15]. The post-mortem examination of 16-year-old dog from the present study that presented with acute neurological signs prior to death, revealed that this dog had a severe CAA, in addition to intraparenchymal Aβ deposits, which are found in AD. In humans, these disorders are correlated with each other, and the prevalence of CAA in AD is over 70% [6]. The severity of CAA has been correlated with progressing AD pathology [16]. In dogs, a condition like AD in humans, with deposition of Aβ plaques has been described [7–12]. Similarly, Aβ deposition within the wall of intraparenchymal and meningeal vessels, resulting in CAA was reported in dogs [7, 10, 11].

In addition to CAA and Aβ deposits in the brain, the dog from the present report had several areas of ischemia/infarction and hemorrhages in the brain. In humans, CAA has been associated with these vascular disorders [6]. In CAA, elastic elements and smooth muscle are replaced by Aβ deposition, which could induce aneurysms and consequently intracerebral hemorrhages [6]. In dogs, intracerebral hemorrhage has been associated with CAA [10].

In the present study, the cerebral ischemic infarcts had a lacunar distribution, demonstrating that intraparenchymal, small and penetrating arteries were the most likely affected vessels. In a previous study, the cause of lacunar infarcts in dogs couldn’t be determined [17]. Ischemic cerebral infarcts have been observed in patients with CAA, which could be a risk factor for cerebral infarcts in aged people [18]. Although the exactly pathogenic mechanism of occurrence of ischemic infarcts associated with CAA is not known [6], Aβ could induce loss of vascular reactivity resulting in a more severe ischemia, with reduction of collateral circulation in ischemic tissues at risk for infarction [19]. In the present case, thickened and hyalinized, intraparenchymal and meningeal vessels that were not immunolabeled for APP were visualized in the dog from the present report. Histologically, these vessels resemble those found in aged people with vascular dementia [20]. Although CAA could contribute for development of ischemic injury in the present case, we cannot rule out other vascular pathological processes. There is an increase evidence of overlapping of AD and cerebrovascular lesions [21]. Vascular changes in the aged brains can be found in a significant proportion of AD patients [20]. Cerebrovascular lesions and AD may coexist in earlier stages of cognitive impairment and may influence its severity and progression, thus making its diagnosis challenging even for neuropathologists [21].

The dog from the present report had cognitive deficits for 1 year such as impaired recognition of human family members and loss of previously learned abilities. Multiple subacute and chronic areas of ischemia in the brain of this dog, as evidenced by astrocytic reactivity within these areas, demonstrates that this dog was suffering from an ongoing cerebrovascular disease. The cognitive deficits presented by this dog are most likely due to Aβ deposition in the brain and neuronal loss due to the multiple old areas of ischemia [15]. However, the dog presented acute neurological signs, and at neurological exam had an ipsilateral postural reaction deficit, ipsilateral circling, and head and neck turn with contralateral head tilt, positional nystagmus, and ataxia. These signs are probably due to the acute ischemic infarct histologically visualized extending from the thalamus to the diencephalon and mesencephalon junction. Similar clinical signs were observed in dogs with midbrain/thalamic infarcts [17].

In conclusion, this report shows a concomitant neurodegenerative disease characterized by APP deposition, which is an Aβ precursor, within the neuroparenchyma and vessels with a severe cerebrovascular disease in an aged dog. Further studies are necessary to elucidate the relationship between vascular disease and Aβ accumulation in the brain of dogs.

## Abbreviations

AD: Alzheimer’s disease
APP: amyloid precursor protein
Aβ: β-amyloid
CAA: cerebral amyloid angiopathy
CNS: central nervous system
CVD: cerebrovascular disease
FMVZ: Department of Pathology of the School of Veterinary Medicine and Animal Science
GFAP: glial fibrillary acidic protein
USP: University of São Paulo
